# Regulation of medicines advertisement in Zimbabwe: an assessment of the impact of pharmaceutical promotion on the prescribing behaviour of healthcare professionals

**DOI:** 10.1186/s12913-021-07399-9

**Published:** 2021-12-14

**Authors:** Tafadzwa Collins Semu, Bernard Ngara, Tinashe Mudzviti

**Affiliations:** Department of Pharmacy & Pharmaceutical Sciences, University of Zimbabwe Faculty of Medicine and Health Sciences, Mazowe Street, Parirenyatwa Complex, P.O Box A178 Avondale, Harare, Zimbabwe

**Keywords:** Pharmaceutical promotion, Regulation, Knowledge, Attitudes, Perception, Healthcare workers

## Abstract

**Background:**

The pharmaceutical industry plays a key role in drug discovery and is considered useful regards to informing the health care workers health care workers about new medicines. Investigations concerning health care workers to industry interactions are less common. The objective of this study is to determine levels of knowledge, attitude and perception towards regulation of pharmaceutical promotion among healthcare practitioners in Zimbabwe.

**Methods:**

A cross-sectional study was used and 330 healthcare practitioners were enrolled into the study. Data collection was done through combined face-to-face interviews and web-based online survey. The relative importance index score and used as a measure of knowledge, attitude and perception levels towards pharmaceutical promotion. Univariate and bivariate analysis was performed using STATA software package.

**Results:**

Our study estimated that 95%, 67%, and 90% of the healthcare practitioners in Zimbabwe have a favourable (i.e. > 65%) relative importance index score of knowledge, attitudes, and perception, respectively, towards regulation of pharmaceutical promotion. Further exploration of the data indicated that the factors that had an association with the knowledge, attitude and perception levels towards regulation of pharmaceutical regulation at 5% level of significance include health care workers’ profession, gender, education level, the nature of the working institution and the number of prescriptions involved per week.

**Conclusion:**

In conclusion, the findings of this study highlighted that in general the health care workers in Zimbabwe have higher levels of knowledge, attitude and perception towards regulation of pharmaceutical promotion, though the attitudes levels weres a  bit lower compared to other domains.

**Supplementary Information:**

The online version contains supplementary material available at 10.1186/s12913-021-07399-9.

## Background

The pharmaceutical industry is among the most important and essential sectors in the world and it continues to expand as time moves on [1]. The pharmaceutical industry plays a key role in drug discovery and is considered useful regards to informing health care workers (HCWs) about medicines [2, 3]. Pharmaceutical companies have shown a tendency to allocate more resources in the form of administration expenditures to the promotion of their products than they spend for drug research and development [4]. Pharmaceutical promotion is defined as information and persuasive activities by the pharmaceutical industry that has an effect to prescription, supply, or use of medicinal drugs. Globally, data shows that the average promotional expenditures for the pharmaceutical industry is estimated to be considerably high around 15–25% while for other industries is around 2% of the total revenues [5]. Different forms of services and favours are offered by the pharmaceutical industry to HCWs that include continued education, conference travel expenses, drug samples, and research grants [6]. Most of the promotional activities are usually done to establish relationships that have a significant impact on HCWs decision-making [4, 7, 8].

HCWs play a pivotal role in provision of proper education on disease and rational therapies to the public [9]. It is generally acceptable that HCWs should be involved in distribution of medicines in a safe and effective manner [10]. HCWs end up getting involved in relationships with the pharmaceutical industries because the HCWs are well positioned to advocate for the patients by speaking through public policy making issues related to the delivery of drug therapy and health care [11]. The pharmaceutical industry is complicated in that the end-user is the patient and not the HCWs, hence the main reason why the pharmaceutical industry recognize that it key to influence the regulatory and practises of HCWs [12]. There are several information resources available to the public such as medical journals and formularies, but most of the HCWs have shown that they depend largely on pharmaceutical promotion, especially in less developed or developing countries where HCWs end up taking inappropriate decisions [13–16]. A systematic review of previous studies in developing countries on the effects of promotion on healthcare practitioners’ behaviour found that at least 50% of the healthcare practitioners had their choice of drug influenced by pharmaceutical promotion [17–21].

The regulation of pharmaceutical promotion is necessary in resolving the conflict between the pharmaceutical industry’s business objectives and that of patients or societal expectations which is to use medicine in the most rational way [22]. However, currently there are existing challenges in regulating their efforts to increase profit especially since pharmaceutical industry is the innovation hub of medicines [23]. The national and international regulations to control pharmaceutical promotional activities were initiated around the 1980s. In Zimbabwe, the Medicines Control Authority of Zimbabwe developed the regulation by adopting the World Health Assembly resolutions of 13th May 1988 on Ethical Criteria for Medicinal Drug Promotion, together with a number of policies adopted by the authority over a number of years. Currently the general requirements for pharmaceutical promotion are governed using Section 35 of the Medicines and Allied Substances Control (General) Regulations, 1991, SI 150 of 1991.

The inappropriate use of medication result in waste of resources, inappropriate patient demand, drug resistance, and increased drug related morbidity and mortality. Many factors contribute to the inappropriate use of medicines, including not only a lack of information but also inaccurate and misleading promotional information. A study conducted in Zimbabwe’s pharmaceutical sector reported that less than 44% of the advertisements listed contraindications, adverse effects, major interactions, warnings and precautions and treatment for overdose, and none had complete information as stipulated by the Medicines and Allied Substances Control Act [24].

While Zimbabwe and many other less developed or developing countries have regulation on pharmaceutical promotion in place, the lack of accurate information on regarding how it affects the healthcare practitioners’ behaviour among other factors, makes it difficult to assess how well pharmaceutical promotion is working. Up to date we have not come upon investigations that seek to determine the healthcare practitioners’ levels of knowledge, attitude and perception (KAP) towards regulation of pharmaceutical promotion, which is the objective of this study. In addition, this study will also explore HCWs characteristics associated with the KAP levels.

## Method

### Data source

The study targeted medical doctors, pharmacists, nurses and, medical/paramedical students practicing in both the public and private institutions of the healthcare sector in Harare, Zimbabwe in the year 2021. A cross-sectional study was applied to enrol study participants in their individual capacity. A structured data collection questionnaire was developed through an extensive review of available literature on KAP of medical professionals regarding regulation of pharmaceutical promotion, and also by reviewing of questionnaires used in similar studies done before. The questionnaire was reviewedby the subject experts for its content and relevance, and their comments were taken into consideration.

In addition to the data on HCWs characteristics, indicators were developed and classified into three main domains to evaluate the KAP levels towards regulation of pharmaceutical promotion separately. Each domain consisted a list of indicators collected on a 5-point Likert scale with responses ranging from strongly disagree-1 to strongly agree-5. The questionnaire was pretested by piloting the tool to 30 participants, and the necessary corrections were made to the final questionnaire based on results of the pilot survey. The final questionnaire was administered face-to-face by trained interviewers and also self-administered by the study participants using an online survey tool which was created in REDCap hosted by the University of Zimbabwe Faculty of Medicine and Health Sciences between May and July 2021. A total of 623 subjects were approached for the web-based survey via e-mails and 180 responded to the survey. A total of 200 subjects were approached for the face-to-face survey and 150 agreed to participate in the survey.

### Data management and analysis

Relative Importance Index (RII) scores calculated for each of the three KAP domains and expressed as a percentage of the maximum possible score. The domain score = (obtained score – minimum possible score)/ (maximum possible score – minimum possible score). In this study, the RII score was further classified favourable if RII is greater or equal to 65%, while less than 65% was classified as unfavourable. We used proportions to summarize qualitative data. We used mean and standard deviation or median and interquartile ranges to summarize quantitative data, where appropriate. We used chi-square test to determine association between both qualitative independent and dependent study variables. We used the t-test or analysis of variance to determine association between qualitative independent and quantitative dependent study variables. Participants with missing response for a particular variable were exclude from any of the analysis that involved that variable. Statistical tests were concluded at 5% level of significance. All data analysis were carried out in STATA software package.

## Results

### Descriptive characteristics of study participants

A total of combined 330 participants responded to both the web-based online and face-to-face survey. Of the total study participants, 30% were medical doctors, 41% were pharmacists, 11% were nurses, and 19% were medical sciences students. The mean age was 36 years. About 63% were male participants. The median working experience was 11 years. 11% were educated up to PhD level, 53% master’s degree, 23% bachelor’s degree. 20% were working at public institutions only, 34% private institutions only and 34% both private and public institutions. The median number of prescriptions per week for the study participants was 50. 67% were above 65% of the aggregated attitude score. 95% were above 65% of the aggregated knowledge score. 90% were above 65% of the aggregated knowledge score.

### Knowledge levels toward pharmaceutical promotion

An estimated 69% agreed that term pharmaceutical promotion refers to marketing of a drug product. 62% are aware of the guidelines for advertising and promotion of medicines in Zimbabwe. 80% agreed that medicines in Zimbabwe cannot be advertised without the approval of the Medicines Control Authority in writing. 86% agreed that the advertisement of any unregistered medicine is not permitted. 78% agreed that a person is not permitted to advertise medicines in connection with any bonus or discount offered. 83% agreed that changes to the approved advertising material should also go through the approval process. 74% agreed that advertisements should not be directed at children. 87% agreed with the assumption that a new product efficacy, quality and safety are similar to the already available medicines. 41% agreed that the pharmacist is not allowed to dispense a different brand of the drug without referring back to the prescriber. 78% agreed that advertisements should claim that a medicine can cure, prevent, or relieve an ailment only if this can be substantiated. 92% agreed that information on adverse reactions, precautions, contraindications and warnings, food and medicine interactions should be available before advertisement. 75% agreed that advertisements to the general public should help people to make rational decisions on the use of medicines.

### Attitude levels toward pharmaceutical promotion

90% support initiatives towards regulation of medicines advertisement. 67% agreed that regulation manages to avoid the pharmaceutical industry from influencing healthcare workers on decisions to prescribe and dispense. 79% agree that regulation reserve rights for patients to be given enough explanations about the reasons for choice of medicines for them. 20% agree that regulation should not allow health care workers in Zimbabwe the right for medicine substitution. 88% agree that regulation improves the ethical and professional behaviour, putting patients first and compliance in the presence of medicines advertisement. 51% agree that regulation has improved the standards for interactions between companies and healthcare practitioners. 30% agree that regulation should avoid companies from sponsorship or support for healthcare practitioners’ attendance at meetings and continuing medical education. 48% agree that regulation should demand acceptable venues and locations for meetings and continuing medical education. 22% agree that regulation should avoid the provision of promotional aids by pharmaceutical industries.

### Perception levels toward pharmaceutical promotion

About 77% agreed that the intensity of promotional activities by medical representatives should be regulated. 83% agreed that health authorities in Zimbabwe should implement policies such that bioequivalence data are mandatory before a product is marketed. About 84% agreed that poor regulation of pharmaceutical promotion may lead to sub-optimal prescription. 86% agreed that poor regulation of pharmaceutical promotion may lead to sub-optimal dispensing. 79% agreed that poor regulation of pharmaceutical promotion may lead to over-expenditure on medicines. 77% agreed that poor regulation of pharmaceutical promotion can influence unjustified medicines regimen switching. 66% agreed that poor regulation of pharmaceutical promotion allows pharmacists to perform medicine substitution without consulting the prescribing physician.

### Factors associated with knowledge towards pharmaceutical promotion

There was an association between aggregated knowledge RII score and profession of the participant (*p* = 0.001), 100% of the pharmacists and nurse had a knowledge RII score above 65%, while 92 and 88% of the medical sciences students and medical doctors, respectively, had a knowledge RII score above 65%. There was an association between aggregated knowledge RII score and gender of the participant (*p* = 0.008), 100% of the female participants had a knowledge RII score above 65%, while 94% of the male participants had a knowledge RII score above 65%. There was an association between aggregated knowledge RII score and education level of the participant (*p* = 0.028), 100% of the participants educated up to PhD or bachelor’s degree had a knowledge RII score above 65%, while 94 and 87% of the participants educated to master’s degree and medical sciences students, respectively, had a knowledge RII score above 65%. There was an association between aggregated knowledge RII score and working institution of the participant (*p* < 0.001), 100% of the participants working at private only or both public and private had a knowledge RII score above 65%, while 88 and 82% of the participants working as students or public sector only, respectively, had a knowledge RII score above 65%. There was no association between aggregated knowledge RII score and age (*p* = 0.1373), work experience (*p* = 0.2136), HCWs involvement in prescriptions (0,214). Additional information is available in supplementary Table 1.

### Factors associated with attitude RII score

There was an association between aggregated attitude RII score and profession of the participant (*p* = 0.016), 71% of the students and nurses had an attitude RII score above 65%, while 72 and 55% of the medical doctors and pharmacists, respectively, had an attitude RII score above 65%. There was an association between aggregated attitude RII score and education level of the participant (*p* = 0.003), 86% of the participants educated up to PhD or masters’ degree had an attitude RII score above 65%, while 77 and 62% of the participants educated to bachelors’ degree and medical sciences students, respectively, had an attitude RII score above 65%. There was an association between aggregated attitude RII score and working institution of the participant (*p* < 0.001), 86% of the participants working at public institutions only had an attitude RII score above 65%, while 76, 75 and 80% of the participants working at both private and public institutions, as medical sciences students or private sector only, respectively, had an attitude RII score above 65%. There was an association between aggregated attitude RII score and the participant involvement in prescriptions (*p* = 0.005), 75% of the participants involved in prescriptions had an attitude RII score above 65%, compared 61% of the participants not involved in prescriptions had an attitude RII score above 65%. There was no association between aggregated attitude RII score and age (*p* = 0.200), gender (*p* = 0.344), work experience (*p* = 0.148), number of prescriptions involved (*p* = 0.2386). Additional information is available in supplementary Table 2.

### Factors associated with perceptions towards pharmaceutical promotion

There was an association between aggregated perception RII score and profession of the participant (*p* < 0.001), 100% of the medical sciences students and nurses had a perception RII score above 65%, while 81 and 89% of the pharmacists and medical doctors, respectively, had a perception RII score above 65%. There was an association between aggregated perception RII score and gender of the participant (*p* = 0.034), 95% of the female participants had a perception RII score above 89%, while 94% of the male participants had a perception RII score above 65%. There was an association between aggregated perception RII score and education level of the participant (*p* = 0.028), 100% of the participants educated up to PhD or are medical sciences students had a perception RII score above 65%, while 92 and 85% of the participants educated to bachelors’ degree and master’s degree, respectively, had a perception RII score above 65%. There was an association between aggregated perception RII score and working institution of the participant (*p* < 0.001), 100% of the medical sciences students had a perception RII score above 65%, while 95, 89 and 75% of the participants working at both public and private sector, public sector only, or public sector only respectively, had a perception RII score above 65%. There was no association between aggregated attitude RII score and age (*p* = 0.305), work experience (*p* = 0.053), HCWs involvement in prescriptions (0,088). Additional information is available in supplementary Table 3.

### Relationship between knowledge, attitude and perception towards pharmaceutical promotion of the study participants

**Fig. 1:**
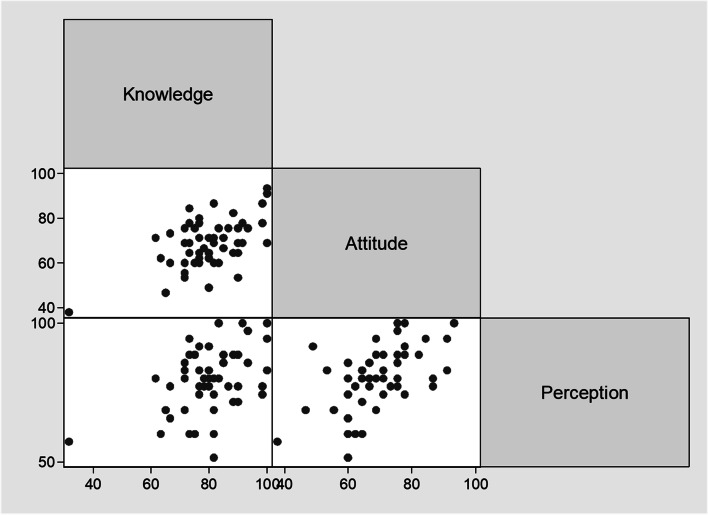
Scatter matrix to determine linear relationship between knowledge, attitude and perception RII scores towards pharmaceutical promotion of the study participants

There was a strong positive linear relationship between knowledge RII score and attitude RII score (spearman correlation coefficient = 0.5, *p* < 0.001). There was a fair positive linear relationship between knowledge RII score and perception RII score (spearman correlation coefficient = 0.3, p < 0.001). There was a strong positive linear relationship between perception RII score and attitude RII score (spearman correlation coefficient = 0.5, p < 0.001). Additional information is available in Fig. 1.

## Discussion

In this study, we estimated the HCWs’ knowledge, attitude and perception levels towards regulation of pharmaceutical promotion. The relative importance index (RII) was calculated as a percentage and used as an aggregate measure for each of the knowledge, attitude and perception domains. To the best of our knowledge, this is the first report to explore HCWs knowledge, attitudes and perceptions towards regulation of pharmaceutical promotion in Zimbabwe using the RII. The RII has been used widely in the past for the purposes of overall ranking of ordinal responses [25–27]. In this study, after the calculation of the RII we used a rule of thumb that participants with a RII score above 65% are more favourable than those with a RII below or equal to 65%. Results from this study estimated that 95, 65 and 90% HCWs have a favourable score towards the KAP RII score, respectively.

The results from this study shows that most of the HCWs in Zimbabwe have high positive knowledge with regards to the regulation of pharmaceutical promotion (i.e.95% had RII > 65%). The result is in the range of with other studies that have estimated a knowledge score towards pharmaceutical promotion of between 50 and 90%. Our further exploration of the data indicated that pharmacists and nurses, female participants, degreed HCWs and participants working at or having a link with private hospitals were associated higher knowledge levels. The variables associated with knowledge score are consistent with results from previous studies that have assessed the factors associated with knowledge levels towards general medical practices [28, 29].

In addition, the results from this study shows that on average the HCWs in Zimbabwe have moderately high positive attitudes towards regulation of pharmaceutical promotion (i.e. 67% had RII > 65%). The result is in the range of with other studies that have estimated the attitude score towards regulation of general medical practises to range between poor to moderate levels [28,30,]. It is quite worrisome that HCWs have significantly negative attitudes towards regulation of promotion having noted reports that the HCWs did not agree to many of the questionnaire indicators meant to assess their attitudes towards regulation of promotion. These observations show that there is a strong need for the improvement of the regulation, monitoring of promotion in Zimbabwe, and probably devising trainings among HCWs to improve their attitudes. Our further exploration of the data indicated that the pharmacists, HCWs educated up to bachelor’s degree, working at private hospitals, and not being involved in prescritions were associated low attitude levels towards regulation of pharmaceutical promotion. Also, similar to knowledge levels, the variables associated with attitude levels are consistent with results from previous studies that have assessed factors associated with the attitude levels towards general medical practices [31–33].

Also, the results obtained from this study shows that most of the HCWs in Zimbabwe have high positive perceptions towards regulation of pharmaceutical promotion (i.e.90% had RII > 65%). The result is in the range of with other studies that have estimated the perception scores towards general medical practises to be high [28, 30]. Our further exploration of the data indicated that the medical nurses and students, female participants, PhD level of education, and working at private hospitals were associated with high levels of perception towards regulation of pharmaceutical regulation. Also, similar to knowledge and attitude levels, the variables associated with perception levels are consistent with results from previous that have assessed factors associated with the attitude levels towards general medical practices [31, 32].

A major limitation to this study is that we the survey response did not reach the minimum 423 subjects required based on the sample size determination procedure due to the time available as stipulated by the university requirements since the research was performed as part of the requirements for fulfilment of a postgraduate degree, hence the marginal effect of the results estimates could be compromised. Additionally, due to the fact that the method of data collection was based on both interviewer administered and self-administered web-based platform, the quality of responses from the web-based platform could have brought additional bias in the event that the survey participants fail to understand some of the questionnaire variables.

## Conclusion

In conclusion, the findings of this study highlighted that HCWs have high positive KAP towards regulation of pharmaceutical promotion, though the attitudes levels was a bit lower when compared to other domains. Policy makers and regulators still have to be concerned about the impact of pharmaceutical promotion and advertisement on HCWs’ behaviours. Continued enforcement of regulations and laws that protect the public from the profit oriented organizations seeking to promote their pharmaceutical products is strongly recommended. Further education and training is recommended to HCWs in order to improve their attitude towards the regulation of pharmaceutical promotion. Future studies should incorporate the views of the policy makers, distributors, patients and other stakeholders with regards to regulation of pharmaceutical promotion.

## Supplementary Information

**ESM 1.**
