# Supplementary material for: Regulation of medicines advertisement in Zimbabwe: an assessment of the impact of pharmaceutical promotion on the prescribing behaviour of healthcare professionals
Source: BMC Health Serv Res. 2021 Dec 14;21:1337. doi: 10.1186/s12913-021-07399-9 (PMC8670253; doi:10.1186/s12913-021-07399-9)
Supplement: Supplementary file 1 — ESM 1. [file 12913_2021_7399_MOESM1_ESM.docx]

Supplementary tables

Supplementary table 1 Factors associated with knowledge towards pharmaceutical promotion

| **Variable** | **Knowledge RII score** | | **p-value** |
| --- | --- | --- | --- |
|  | **<65** | **>=65** |  |
| Profession, n (%) |  |  |  |
| Medical doctor | 10 (12) | 75 (88) | 0.001 |
| Pharmacist | - | 115 (100) |  |
| Nurse | - | 35 (100) |  |
| Student | 5 (8) | 55 (92) |  |
| Age in years, mean (SD) | 34 (6) | 37 (10) | 0.1373 |
| Gender, n (%) |  |  |  |
| Male | 10 (6) | 170 (94) | 0.008 |
| Female | - | 110 (100) |  |
| Work experience in years, mean (SD) | 11 (0.5) | 13 (80.8) | 0.2136 |
| Education level, n (%) |  |  |  |
| Student | 5 (13) | 35 (87) | 0.028 |
| Bachelors | - | 60 (100) |  |
| Masters | 10 (6) | 150 (94) |  |
| PhD | - | 35 (100) |  |
| Other | - | 5 (100) |  |
| Working institution, n (%) |  |  |  |
| Public | 10 (18) | 45 (82) | <0.001 |
| Private | - | 105 (100) |  |
| Both | - | 95 (100) |  |
| Student | 5 (13) | 35 (88) |  |
| Involved in prescriptions, n (%) |  |  |  |
| Yes | 5 (4) | 135 (96) | 0.214 |
| No | 10 (6) | 150 (94) |  |

SD- Standard deviation; IQR- Interquartile Range

Supplementary table 2 Factors associated with attitudes towards pharmaceutical promotion

| **Variable** | **Attitude RII score** | | **p-value** |
| --- | --- | --- | --- |
|  | **<65** | **>=65** |  |
| Profession, n (%) |  |  |  |
| Medical doctor | 25 (28) | 65 (72) | 0.016 |
| Pharmacist | 50 (45) | 60 (55) |  |
| Nurse | 10 (29) | 25 (71) |  |
| Student | 15 (25) | 45 (71) |  |
| Age in years, mean (SD) | 35 (6) | 36 (11) | 0.200 |
| Gender, n (%) |  |  |  |
| Male | 60 (32) | 130 (68) | 0.344 |
| Female | 30 (29) | 75 (71) |  |
| Work experience in years, mean (SD) | 12 (.6) | 13 (0.7) | 0.148 |
| Education level, n (%) |  |  |  |
| Student | 15 (38) | 25 (62) | 0.003 |
| Bachelors | 15 (23) | 50 (77) |  |
| Masters | 5 (14) | 30 (86) |  |
| PhD | 5 (14) | 30 (86) |  |
| Other | - | 5 (100) |  |
| Working institution, n (%) |  |  |  |
| Public | 10 (18) | 48 (82) | <0.001 |
| Private | 50 (50) | 50 (50) |  |
| Both | 25 (24) | 80 (76) |  |
| Student | 10 (25) | 30 (75) |  |
| Involved in prescriptions, n (%) |  |  |  |
| Yes | 35 (25) | 105 (75) | 0.005 |
| No | 65 (39) | 100 (61) |  |

SD- Standard deviation; IQR- Interquartile Range

Supplementary table 3 Factors associated with perception towards pharmaceutical promotion

| **Variable** | **Perception RII score** | | **p-value** |
| --- | --- | --- | --- |
|  | **<65** | **>=65** |  |
| Profession, n (%) |  |  |  |
| Medical doctor | 10 (11) | 85 (89) | <0.001 |
| Pharmacist | 20 (19) | 85 (81) |  |
| Nurse | - | 30 (100) |  |
| Student | - | 60 (100) |  |
| Age in years, mean (SD) | 35 (4) | 36 (10) | 0.305 |
| Gender, n (%) |  |  |  |
| Male | 20 (11) | 155 (89) | 0.034 |
| Female | 5 (5) | 105 (95) |  |
| Work experience in years, mean (SD) | 10 (4) | 13 (4.5) | 0.053 |
| Education level, n (%) |  |  |  |
| Student | - | 40 (100) | 0.006 |
| Bachelors | 5 (8) | 55 (92) |  |
| Masters | 25 (15) | 140 (85) |  |
| PhD | - | 30 (100) |  |
| Other | - | 5 (100) |  |
| Working institution, n (%) |  |  |  |
| Public | 15 (25) | 45 (75) | <0.001 |
| Private | 10 (11) | 85 (89) |  |
| Both | 5 (5) | 95 (95) |  |
| Student | - | 40 (100) |  |
| Involved in prescriptions, n (%) |  |  |  |
| Yes | 10 (7) | 130 (93) | 0.088 |
| No | 20 (13) | 140 (87) |  |

SD- Standard deviation; IQR- Interquartile Range
